# The relationship of school performance with self-control and grit is strongly genetic and weakly causal

**DOI:** 10.1038/s41539-023-00198-3

**Published:** 2023-12-04

**Authors:** Sofieke T. Kevenaar, Elsje van Bergen, Albertine J. Oldehinkel, Dorret I. Boomsma, Conor V. Dolan

**Affiliations:** 1 Department of Biological Psychology, Vrije Universiteit Amsterdam, Van der Boechorststraat 7, 1081 BT Amsterdam, The Netherlands; 2 Research Institute LEARN!, Vrije Universiteit Amsterdam, Amsterdam, the Netherlands; 3 Amsterdam Public Health Research Institute, Amsterdam, the Netherlands; 4 University of Groningen, University Medical Center Groningen, Groningen, the Netherlands; 5 Amsterdam Reproduction and Development Research Institute, Amsterdam, the Netherlands

**Keywords:** Human behaviour, Education, Human behaviour

## Abstract

The non-cognitive skills self-control and grit are often considered predictors of school performance, but whether this relationship is causal remains unclear. We investigated the causality of this association using a twin design. Specifically, we evaluated the direct impact of self-control and grit on school performance, while controlling for genetic or environmental influences common to all three traits (i.e., confounding). Teachers of 4891 Dutch 12-year-old twin pairs (of which 3837 were complete pairs) completed a survey about school performance (school grades), self-control (ASEBA self-control scale), and the perseverance aspect of grit. Our analysis aimed to determine the direct impact of self-control and grit on school performance, while simultaneously controlling for genetic or environmental confounding. Establishing the regression relationship corrected for confounding supports the interpretation of the regression relationship as causal. In all analyses, we corrected for sex, rater bias of the teachers, and parental socioeconomic status. Initially, in the standard regression, self-control, and grit explained 28.4% of the school performance variance. However, allowing for genetic confounding (due to genetic pleiotropy) revealed that most of this association could be attributed to genetic influences that the three traits share. In the presence of genetic pleiotropy, the phenotypic regression of school performance on self-control and grit accounted for only 4.4% (i.e., the effect size association with the causal hypothesis). In conclusion, self-control and grit predict school performance primarily due to genetic pleiotropy, with a much smaller causal effect (*R*^2^ = 4.4%). This suggests that interventions targeting self-control and grit alone may yield limited improvements in school performance.

## Introduction

Much research has focused on self-control and grit as predictors of school and academic performance. Grit comprises consistency of interest and perseverance, and self-control is the ability to self-regulate conflicting impulses (Duckworth et al., 2016). Perseverance and self-control have generally been found to be associated with school and academic performance^1–7^. For instance, Lam & Zhou^5^ reported an average correlation of 0.17 between grit and school performance in school children (based on 56 correlations). The average correlation was 0.14 in students in higher education (based on 60 effect sizes; see also Fernández Martín et al.^8^). In a recent study of Czech school children, Vazsonyi, et al.^9^ found that self-control predicted school performance (both teacher-rated and grades) while controlling for motivation and intelligence. In a twin study, Kevenaar et al.^10^ found that self-control and grit together explained 28.4% of the variance in school performance. The decomposition of the phenotypic regression relationship into genetic and environmental components revealed that the phenotypic associations were mainly due to genetic influences common to self-control, grit, and school performance.

While it is well established that self-control and grit predict academic outcomes, most studies tend not to address causality^2,11,12^. One of the few studies that provide a basis for a causal interpretation regarding self-control was conducted by Duckworth et al.^13^, who showed that within-individual changes in self-control over time predict changes in academic achievement, but not vice versa, which suggests a causal effect only from self-control to achievement. A small (*N* = 53) intervention study on self-regulation indicated that self-regulation training affected math performance, which is also consistent with a causal effect of self-regulation^14^. Regarding grit, Jiang et al.^15^ followed 193 children longitudinally, and found reciprocal effects between grit and academic achievement, consistent with a reciprocal causal relation. Achievement was found to related to the perseverance, but not to the consistency facet of grit. Postigo et al.^16^ studied a large sample of children (*N* = 5371) longitudinally from age 10 to 14. They reported an effect of grit on school performance (grades) in a two-occasion panel model, which is consistent with a causal model. Hence, as far as it has been studied, most research suggests a causal effect of self-control and grit on school performance, rather than the other way around.

The interpretation of the effects of self-control and grit on school performance as causal is appealing, as it is plausible that these non-cognitive factors facilitate school or academic performance. However, more research, employing different designs, is needed to establish causal pathways, and to rule out possible—correlational, non-causal—sources of association. Such non-causal sources may be both genetic and environmental. For instance, the association may be due to common genetic influences (pleiotropy: the same genes affect multiple traits; MacKay^17^), or due to a rearing environment that is conducive to both cognitive and non-cognitive influences on school performance. It is also important to take note of the challenging possibility that causal and non-causal accounts of the associations are not mutually exclusive.

In contrast to our previous work, which addressed prediction (Kevenaar et al.^10^) the current study addressed causation. Kevenaar et al.^10^ examined the phenotypic regression relationship of self-control and grit with school performance, and decomposed these into genetic and environmental components. Thus, Kevenaar et al.^10^ focussed on the genetic and environmental contributions to the prediction of school performance, but did not address causality. In contrast, the present focus is on establishing the direct phenotypic effects of self-control and grit on school performance, corrected for genetic or environmental confounding. Demonstrating the direct phenotypic regression relationships corrected for confounding supports the interpretation of these relationships as causal. In the current article, we analyze the same cohort data, but fitted causal models.

The classical twin design provides a means to estimate the putatively causal phenotypic relationships between the non-cognitive factors and school performance while accounting for the traits’ background genetic and environmental correlations. Kohler et al.^18^ and McAdams et al.^19^ provided detailed discussions of causal modelling based on the classical twin design. Pingault et al.^20^ discuss more generally the role of genetic data in causal inference using observational data (including twin data). For a recent application of this approach in the study of alcohol use and anxiety, we refer to Torvik et al.^21^. For applications in educational sciences, we refer to van Bergen et al.^22,23^ and Malanchini et al.^24^ who studied the causal effects between literacy skills and literacy enjoyment. Using causal modelling in the classical twin design, we investigated in the current work the causal relationship between teacher-rated self-control, grit, and school performance, in the presence of possible genetic and environmental confounding, that is, non-causal associations that are attributable to genetic and environmental influences common to the phenotypes.

The outline of this article is as follows. First, we introduce the statistical model based on the classical twin design, and provide a summary of the results of Kevenaar et al.^10^. Next, we present a causal twin model, which we apply to explore the putative causal influence of self-control and grit and school performance. Then, we present the results of fitting the causal twin model including genetic confounding (a.k.a. genetic pleiotropy), and environmental confounding.

The twin design is a genetically informative design, which is applied to decompose phenotypic variance and covariance into genetic and environmental components. With respect to the environmental components, we distinguish shared (C) and unique environmental (E) variance. The latter (E) is unique to the individual twins, not shared, and, as such, contributes to the phenotypic variance, but not the phenotypic covariance (resemblance) of the twins. Shared environmental variance originates in environmental influences that twins share and contributes to the phenotypic covariance of the twins. With respect to the genetic components, we can distinguish additive genetic (A) variance and dominance (D) variance, where the former is due to the additive (linear) effects of alleles, and the latter is due to non-additive effects of alleles within relevant genetic loci on the phenotype^25^. Because monozygotic (MZ) twins are genetically (nearly) identical, both A and D contribute 100% to the MZ phenotypic covariance. Dizygotic (DZ) twins, like full sibs, on average share 50% of their alleles, as inherited from their biological parents. Based on allele sharing, we expect 50% of the additive genetic variance to contribute to the DZ phenotypic covariance. The dominance variance attributable to a given locus contributes to the phenotypic resemblance, only if the twins are genetically identical by descent at the locus. Considering, as an example, a diallelic locus with alleles B and b, 25% of the DZ twins are genetically identical (i.e., both BB, Bb, or bb). Therefore, we expect 25% of the dominance variance to contribute to the DZ phenotypic covariance. When fitting the classical twin model to data from MZ and DZ twin pairs to identify the variance components, we need to limit the number of components to three, that is, an ADE or an ACE model. The choice is usually based on the following rule of thumb concerning the phenotypic twin correlation, *r*_mz_ and *r*_dz_.

1 $${r}_{{\rm{mz}}} > 2* {r}_{{\rm{dz}}}$$

2 $${r}_{{\rm{mz}}} < 2* {r}_{{\rm{dz}}}$$

If Eq. (1) holds, it suggests an ADE model, and if Eq. (2) holds, it suggests an ACE model^26,27^ Based on our earlier work on the same data^10^, we fitted an ADE model to all three phenotypes (i.e., self-control, grit, and school performance) and decomposed the 3 × 3 covariance matrix Σ_Ph_ as follows:

3 $${\Sigma }_{{\rm{Ph}}}={\Sigma }_{{\rm{A}}}+{\Sigma }_{{\rm{D}}}+{\Sigma }_{{\rm{E}}}$$

This decomposition (Eq. 3) is achieved by modelling the 6 × 6 MZ and DZ twin covariance matrices. The matrices are 6 × 6, because of the three phenotypes for both twin 1 and twin 2 (the first and second born, for example), as in Table 1. The MZ and DZ twin covariance matrices Σ_Ph|MZ_ (4)and Σ_Ph|DZ_ (5) are as follows:

4 $$\begin{array}{rcl}&{\text{MZ twin 1}}\qquad\qquad\quad {\text{MZ twin 2}}\\{\sum}_{Ph|MZ}=\begin{array}{c}{\text{MZ twin 1}}\\ {\text{MZ twin 2}} \end{array}&\left[\begin{array}{cc} \sum_{\rm{A}} +\sum_{\rm{D}}+ \sum_{\rm{E}} & \sum_{\rm{A}}+ \sum_{\rm{D}}\\ \sum_{\rm{A}}+ \sum_{\rm{D}} & \sum_{\rm{A}} +\sum_{\rm{D}}+ \sum_{\rm{E}}\end{array}\right]\end{array}$$

**Table 1:** MZ and DZ correlation matrices of school performance (SP), self-control (SC), and Grit, conditional on fixed sex and SES effects, and random rater effects, and corrected for censoring.

The correlations shown in dark blue represent the within-person correlations between traits, which are expected to be similar in MZ and DZ. The correlations shown in bold represent the within-trait twin correlations. These are higher in MZ than DZ, suggesting genetic influences on the traits. The correlations shown in light blue represent the cross-trait, cross-twin correlations (e.g., the correlation between school performance of one twin and self-control of the cotwin). These are higher in MZ than DZ, suggesting genetic correlations between the traits. 1 = Twin 1; 2 = Twin 2.

And

5 $$\begin{array}{rcl}&{\text{DZ twin 1}}\qquad\qquad\quad {\text{DZ twin 2}}\\{\sum}_{Ph|DZ}=\begin{array}{c}{\text{DZ twin 1}}\\ {\text{DZ twin 2}} \end{array}&\left[\begin{array}{cc} \sum_{\rm{A}} +\sum_{\rm{D}}+ \sum_{\rm{E}} & \frac{1}{2}\sum_{\rm{A}}+ \frac{1}{4}\sum_{\rm{D}}\\ \frac{1}{2}\sum_{\rm{A}}+ \frac{1}{4}\sum_{\rm{D}} & \sum_{\rm{A}} +\sum_{\rm{D}}+ \sum_{\rm{E}}\end{array}\right]\end{array}$$

The covariance matrices Σ_A_, Σ_D_, and Σ_E_ may be subject to various parameterizations, depending on computational or substantive considerations (see below).

**Fig. 1:**
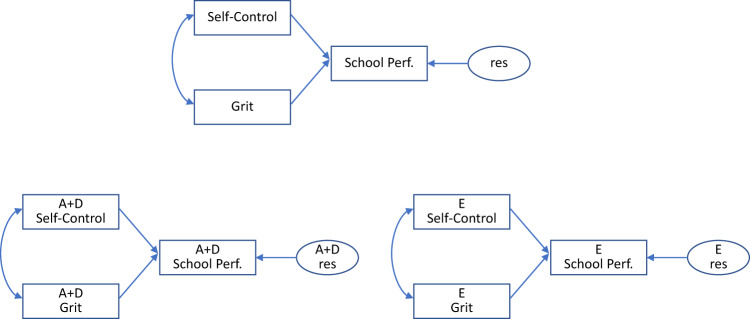
The phenotypic regression model and the decomposed regression models. Top panel: the phenotypic regression model. The regression residual is denoted *res*. Bottom panel: the A + D regression model and the E regression model. These E and A + D models decompose the phenotypic regression results into A + D (based on Σ_A_ + Σ_D_) and unshared environmental E regression results (based on Σ_E_). The covariates (SES, sex, and rater) are not depicted.

As reported in Kevenaar et al.^10^, we previously analyzed teacher ratings of self-control, grit, and school performance in MZ and DZ twins using the same data that were analyzed for the present article. The results were obtained with a correction for the main effects of sex and SES, and a correction for the rater (i.e., the teacher of the twins). Given the ceiling effect in the distribution of the data (see below), we fitted ADE models using maximum likelihood estimation with a correction for right-censoring (see also de Zeeuw et al.^28^). First, school performance was regressed on self-control and grit using linear regression analysis of these observed variables. Second, the regression analyses were conducted at the broad-sense genetic level (Σ_A_ + Σ_D_), and at the unshared environmental level (Σ_E_). Because self-control and grit are correlated (about 0.65 in the present data), the decomposition of school performance variance (conditional on the covariates) comprised four variance components: a component due to self-control, a component due to grit, a component involving the covariance between self-control and grit, and the residual variance component. At the level of the phenotypic regression model (see Fig. 1 top), self-control and grit explained 28.4% of the school performance variance, with the following decomposition: 4.4% due to self-control, 13.0% due to grit, and 10.9% involving the covariance of self-control and grit). Considering the unique contributions of self-control and grit, grit emerged as the stronger predictor (13% vs 4.4%).

Subsequently, the ADE model was fitted to the twin data, and the regression analyses were conducted twice: once at the level of Σ_A_ + Σ_D_ (the broad-sense genetic covariance matrix) and once at the level of Σ_E_ (the unshared environmental covariance matrix) (see Fig. 1, bottom two panels). The results showed that the phenotypic decomposition of school performance variance was largely attributable to broad-sense genetic factors. Thus, the phenotypic regression relationship between the predictors self-control and grit and school performance was largely a reflection of common genetic influences.

The results of the regression analyses, both at the phenotypic level and at the genetic and environmental level, are consistent with a causal model, but do not prove causality. This is because correlation—established at the phenotypic level or the genetic and environmental level—does not imply causation. Below we present a causal twin model that addresses causality by fitting the phenotypic regression model, while accounting for the possibility of genetic or environmental background correlation (i.e., genetic or environmental confounding^29^).

**Fig. 2:**
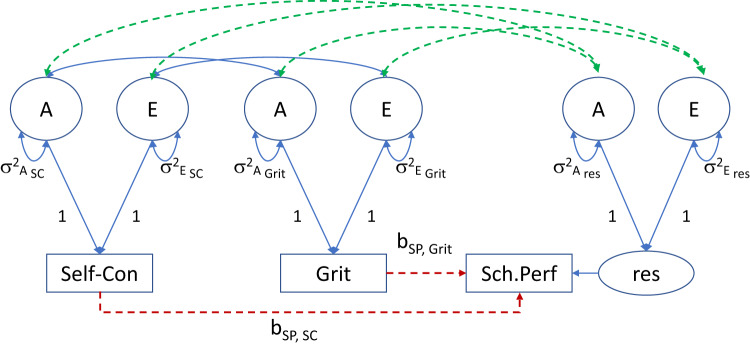
The causal model. The causal model with background A, D (not shown), and E correlations is represented by dashed double-headed arrows, shown in green. SP school performance, SC self-control. The parameters b_SP,SC_ and b_SP,Grit_, shown in red, are the causal regression coefficients. The residual in the regression of SP on SC and Grit is denoted *res*. To avoid clutter, the Ds (and for each phenotype) and the covariates (SES, sex, and rater) are not depicted.

In the current paper, we fit a causal twin model. The causal twin model is depicted in Fig. 2. This model allows us to assess the putative causal regression relationships, while taking into account A, D, or E background correlation, i.e., A, D, or E confounding^18,20,30–33^. In Fig. 2, the background A, D, and E correlations are represented by the dashed double-headed arrows. In this approach, the strongest support for the causal hypothesis would be the finding that the phenotypic regression coefficients (denoted b_SP,SC_ and b_SP,Grit_) are significant, while the background correlations are all zero. This would support causality in that the results then demonstrate the phenotypic regression relations between the predictors and school performance are not due to background (A, D, or E) confounding, but to the direct phenotypic, putatively causal, relations. The causal model is refuted if the regression coefficients are zero in the presence of A, D, and/or E background correlations, as this means that the associations between self-control and grit and the dependent variable school performance are not due to direct, causal relations. Rather, they are attributable to environmental or genetic influences common to the three phenotypes (see Kohler et al.^18^ for a detailed treatment of this and related twin models). Note that the finding that the parameters b_SP,SC_ and b_SP,Grit_ (Fig. 2) differ significantly from zero does not rule out A, D, or E confounding. As mentioned above, the direct (phenotypic) causal effects and confounding are not mutually exclusive. In this causal twin model, we explore this possibility by fitting the phenotypic regression model, while allowing for A, D, or E confounding. We do this by including the dashed double-headed arrows in the model (Fig. 2). We modelled data of 8728 twin children from the Netherlands Twin Register. The twins’ grit, self-control, and school performance were rated by their teacher when they were about 12 years old.

## Results

### Descriptives

**Fig. 3:**
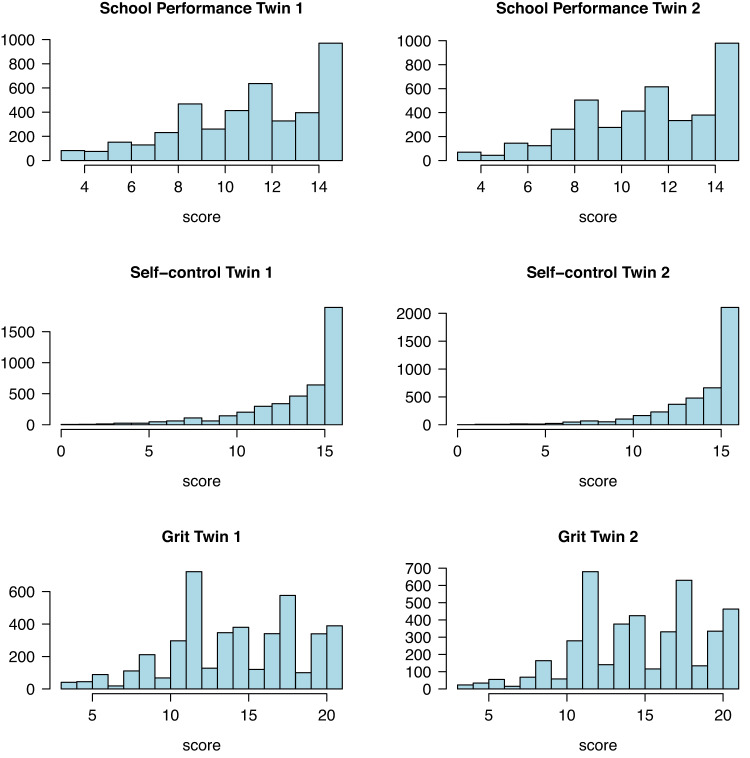
Histograms. Histograms of the raw data of the first twin members and the second members (MZ and DZ twins pooled).

Histograms of the raw data are given in Fig. 3. The right censoring (ceiling effects) is evident in all three phenotypes. In the MZ and DZ twin 1 members, the skewnesses equal −0.63 (school performance), −1.63 (self-control), and −0.31 (grit); in the MZ and DZ twin 2 members, these equal −0.57, −1.93, and −0.48, respectively. The estimates of the MZ and DZ correlation matrices, based on the saturated model, are given in Table 1 (these are conditional on the covariates sex, SES, and rater, and corrected for censoring).

The MZ twin correlations in Table 1 for school performance, self-control, and grit equal 0.809 (95% CIs: 0.788–0.928), 0.715 (95% CIs: 0.685–0.716), and 0.751 (95% CIs: 0.722–0.778), respectively. The DZ twin correlations equal 0.419 (95% CIs: 0.376–0.460), 0.276 (95% CIs: 0.250–0.289), and 0.176 (95% CIs: 0.114—0.234), respectively. The twin correlations of self-control and grit suggest an ADE model (*r*_MZ_ > 2*r_DZ_). The twin correlations of school performance suggest an AE model, but the 95% CIs do not rule out the possibility of an ADE model (see Keller & Coventry^26^). The correlations between self-control and grit are about 0.72. The correlations between school performance on the one hand and self-control or grit on the other hand range from 0.458 to 0.523.

### Phenotypic regression model (Model 1)

We fitted the phenotypic regression model in OpenMx, correcting for the family clustering of the data (i.e., MZ and DZ twins in pairs). The aim of this is to obtain regression results of regressing school performance of self-control and grit at the population level. We decomposed the proportion of explained variance of school performance (i.e., R^2^ statistic), into the part due to self-control, the part due to grit, and the part that involves the covariance of self-control and grit. Because the third part involves covariance, it cannot unambiguously be attributed to either self-control or grit. As reported by Kevenaar et al., (2023), we found that self-control and grit explained 28.4% of the variance in school performance (*R*^2^ = 0.284). The unique contributions of self-control and grit equalled 4.4% (95% CIs: 2.07%—7.91%) and 13.0% (95% CIs: 8.03%–19.48%), respectively. The remaining 10.9% was a function of the covariance of the predictors (95% CIs: 9.08%–12.43%). The regression coefficients equalled b_SP,SC_ = 0.191 (95% CIs: 0.132–0.251) and b_SP,Grit_ = 0.331 (95% CIs: 0.252–0.412). From these results, grit emerges as the stronger predictor. Based on the results in Table 2, we found the predictors (self-control and grit) are correlated 0.71 on average. We checked whether this correlation resulted in multicollinearity by calculating the variance inflation factor (VIF^34^). The VIF associated with the predictors was about 2.06. The rule of thumb concerning the interpretation varies considerably, with VIF > 2.5 to VIF > 10 signalling multicollinearity. As the present value is about 2.06, we conclude that multicollinearity is not an issue here.

**Table 2:** Covariance (cov) matrices, correlation (cor) matrices; and proportions, based on the ADE twin model.

	Phenotypic cov matrix			Phenotypic cor matrix					
Σ_Ph_	SP	SC	GRIT	SP	SC	GRIT			
SP	11.725	6.207	6.615	1.000	0.474	0.502	-		
SC	6.207	14.616	10.565	0.474	1.000	0.718	-	-	
GRIT	6.615	10.565	14.824	0.502	0.718	1.000	-	-	-

	A cov matrix			A cor matrix			proportions Σ_A_/Σ_Ph_		
Σ_A_	SP	SC	GRIT	SP	SC	GRIT	SP	SC	GRIT
SP	9.009	4.806	4.868	1.000	0.653	0.996	**0.768**	0.774	0.736
SC	4.806	6.006	2.852	0.653	1.000	0.715	0.774	**0.411**	0.270
GRIT	4.868	2.852	2.649	0.996	0.715	1.000	0.736	0.270	**0.179**

	D cov matrix			D cor matrix			proportions Σ_D_/Σ_Ph_		
Σ_D_	SP	SC	GRIT	SP	SC	GRIT	SP	SC	GRIT
SP	0.288	0.587	1.220	1.000	0.543	0.792	**0.025**	0.095	0.184
SC	0.587	4.054	5.449	0.543	1.000	0.943	0.095	**0.277**	0.516
GRIT	1.220	5.449	8.239	0.792	0.943	1.000	0.184	0.516	**0.556**

	E cov matrix			E cor matrix			proportions Σ_E_/Σ_Ph_		
Σ_E_	SP	SC	GRIT	SP	SC	GRIT	SP	SC	GRIT
SP	2.428	0.813	0.527	1.000	0.244	0.170	**0.207**	0.131	0.080
SC	0.813	4.557	2.264	0.244	1.000	0.535	0.131	**0.312**	0.214
GRIT	0.527	2.264	3.936	0.170	0.535	1.000	0.080	0.214	**0.265**

The results are conditional on the sex and SES effects, and the random rater (teacher) effects. The values shown in bold are the standardized variance components. For instance for school performance, 76.8% is attributable to additive genetic factors (i.e., the narrow-sense heritability is 0.768), 2.5% is attributable to genetic dominance factors (i.e., the broad-sense heritability is 0.793), and 20.7% is attributable to non-shared environmental influences and measurement error.

### ADE twin model (Model 2)

The 3×3 covariance matrices Σ_A_, Σ_D_, and Σ_E_ were parameterized using lower triangle matrices (i.e., the Cholesky decomposition^30^):

6 $${\sum }_{{\rm{A}}}={\Delta }_{{\rm{A}}}{\Delta }_{{{\rm{A}}}^{{\rm{t}}}}$$

7 $${\sum }_{{\rm{D}}}={\Delta }_{{\rm{D}}}{\Delta }_{{{\rm{D}}}^{{\rm{t}}}}$$

8 $${\sum }_{{\rm{E}}}={\Delta }_{{\rm{E}}}{\Delta }_{{{\rm{E}}}^{{\rm{t}}}}$$

In Eqs. (6), (7), and (8), Δ_A_, Δ_D_, and Δ_D_ are 3 × 3 lower triangular matrices. The results of fitting the ADE twin model are given in Table 2.

The right columns of Table 2 show the proportions of the phenotypic variances and covariances attributable to A, D, and E factors. These proportions provide an interpretable decomposition of phenotypic (co)variance. For instance, the standardized variance of grit, conditional on the covariates (SES, sex, and rater), is expressed in proportions as follows 0.179 (A), 0.556 (D), and 0.265 (E). So, we know that about 73% of the phenotypic variance is due to genetic effects (17.9% + 55.6%). The phenotypic correlation between school performance and grit is 0.502. This correlation is expressed as proportions 0.736 (A), 0.184 (D), and 0.080 (E). So, 8% of the phenotypic correlation is attributable to E, unshared environmental factors, and 92% (73.6% + 18.4%) is attributable to genetic factors). The correlation matrices are displayed in the middle columns. So, for example, the additive genetic correlation between self-control and grit is 0.715. The results in Table 2 are conditional on the covariates sex, SES, and rater (teacher). As mentioned above, the rater effect was modelled as a random effect, i.e., part of the covariance structure. Table 3 contains the standardized variance components including the proportion attributable to the rater effect. Table 3 includes the estimates of the covariance matrices Σ_A_, Σ_D_, and Σ_E_, the phenotypic covariance matrix Σ_Ph_ (i.e., Σ_A_ + Σ_D_ + Σ_E_), and the associated correlation matrices (i.e., Σ_A_, Σ_D_, Σ_E_, and Σ_Ph_ standardized).

**Table 3:** ADE standardized variance components (corrected for sex and SES), including the variance attributable to rater (95% CIs in parentheses).

	A	D	E	Rater (Teacher)
School Performance	0.712 (0.609–0.757)	0.022 (0.003–0.105)	0.192 (0.183–0.221)	0.073 (0.044 –0.011)
Self–control	0.410 (0.261–0.538)	0.276 (0.207–0.374)	0.311 (0.286–0.315)	0.002 (0.000–0.113)
Grit	0.139 (0.093–0.226)	0.437 (0.371–0.512)	0.209 (0.194–0.231)	0.212 (0.183–0.250)

The four variance components are standardized, so add up to 1.

The standardized A component gives narrow-sense heritability and the standardized A component + the standardized D component gives the broad-sense heritability.

The standardized variance of grit, conditional on the covariates (SES, sex, and rater), is expressed as proportions as follows: 0.139 (A), 0.437 (D), 0.209 (E), and 0.212 (rater). We note that the rater (teacher) effects, in terms of standardized variance are quite variable, ranging from 21.2% (grit) to 0.2% (self-control).

### Causal regression model without confounding (Model 3)

The causal regression model is depicted in Fig. 2. In this model, the background correlations (associated with the dashed double-headed arrows in Fig. 2) are fixed to zero, meaning that there is no background correlation due to common A, D, or E influences (i.e., no confounding). As such, this model is consistent with the causal hypothesis that self-control and grit are causes of school performance. The LRT of this model relative to the ADE model equals LRT = 155.8, *df* = 4 (*p* < 0.008). Table 4 includes an overview of the LRTs and Akaike information criterion (AIC). The AIC is a goodness of fit measure that balances model complexity and model fit to achieve a parsimony-related fit statistic. In theory, the model with the lowest AIC is the model of choice.

**Table 4:** Model fit comparison tests, with the preferred model printed in bold.

Model	Δ*df*	ΔLL	AIC	*p* value
ADE			105451.9	
Causal ADE	4	155.8	105599.7	<0.001
ADE			105451.9	
**Causal ADE** + **A confounding**	**2**	**0.407**	**105448.3**	**0.815**
ADE			105451.9	
Causal ADE + E confounding	2	24.8	105472.7	<0.001
ADE			105451.9	
Causal ADE + D confounding	2	38.4	105486.3	<0.001

Δ*df* is the difference of degrees of freedom of the models, ΔLL is the difference in the minus 2 log-likelihood of the models, AIC is the Akaike information criterion. The preferred model is printed in bold.

The ADE model includes six parameters to model the phenotypic covariance between self-control and school performance and grit and school performance (two A covariances, two D covariances and two E covariances). But the causal model includes two parameters to model these covariances (i.e., the regression coefficients b_SP, SC_ and b_SP, Grit_). The difference in the number of parameters, which equals the degrees of freedom, is four. The LRT (155.8, *df* = 4, *p* < 0.008) clearly indicates that the causal model, without confounding, does not fit well, relative to the ADE model. The AIC value of this model (i.e., 105599.7) is also the largest in Table 4. This suggests at least that the effects of the predictors self-control and grit on the outcome school performance are not purely causal.

### The causal model with confounding (Models 4, 5, 6)

**Fig. 4:**
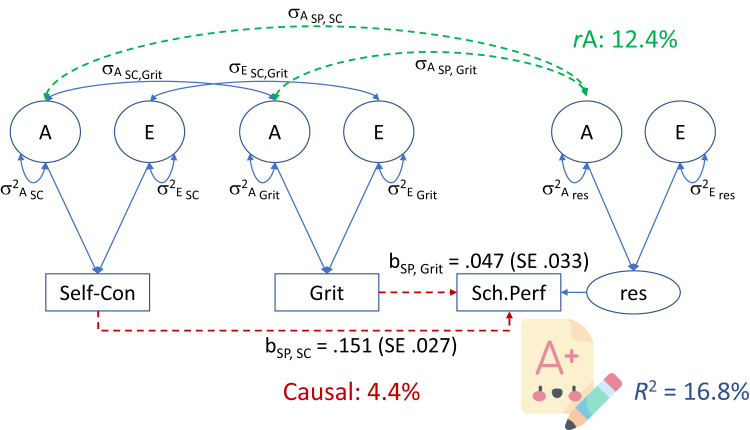
The model of choice. The model of choice shows direct A confounding (in green; dashed double-headed arrows with covariances σ_A SP,Grit_, σ_A SP,SC_) and causal effects (in red; parameters b_SP,SC_ and b_SP,Grit_). 16.8% of the variance in school performance is explained, for a large part due to genetic confounding (12.4%) and a small part due to the causal effects (4.4%), driven by the effect of self-control. To avoid clutter, the Ds (i.e., dominance genetic variance) and covariates are not depicted. A additive genetic variance, E non-shared environmental variance and measurement error, SC self-control, SP school performance, res residual.

We added A, D, and E confounding to the model by including the relevant background A, D, and E correlations (dashed double-headed arrows in Fig. 2). We considered A, D, and E confounding consecutively. We did not consider more than one source of confounding, as this, in combination with the phenotypic regression coefficients, renders the model equivalent to the ADE model in terms of the number of parameters used to model the associations. The LRT statistics, based on the comparison of the ADE model with the causal model with confounding, are LRT = 0.407, *df* = 2, *p* = 0.815, LRT = 24.8, df = 2, *p* < 0.0001, and LRT = 38.4, *df* = 2, *p* < 0.001, given A, E, and D confounding, respectively. The tests have two degrees of freedom, because the ADE model includes six parameters to model the phenotypic covariance between self-control and school performance and grit and school performance (two A covariances, two D covariances and two E covariances). The causal regression model with confounding does this with four parameters: the regression coefficients and two A, D, or E covariances. The LRTs suggest that the causal model with A confounding fits the data well, relative to the ADE model (*Χ*^2^_Δ_ (2) = 0.407, *p* = 0.81), but the other models clearly do not. The AIC also identifies the causal model with A confounding as the best fitting model, as this model has the lowest AIC value (i.e., 105448.3). The model of choice with direct causal effects and A confounding is shown in Fig. 4.

In the causal model with A confounding, the estimates of the causal regression coefficients b_SP,SC_ and b_SP,Grit_ are 0.151 (s.e. 0.027) and 0.047 (s.e. 0.033), respectively. The LRT statistics of the tests of b_SP,SC_ = 0 and b_SP,Grit_ = 0 are 2.44, *df* = 1, *p* = 0.118 (b_SP,SC_) and 29.18, *df* = 1, *p* < 0.001 (b_Grit,SC_). While the test of b_SP,SC_ is not statistically significant, we retained this parameter in the model, and in the calculation of components of variance of school performance. The decomposition of the variance of school performance in raw and standardized variance components is given in Table 5.

**Table 5:** Decomposition of the school performance variance in raw and standardized estimates with 95% confidence intervals (95% CIs) in the causal regression model with A confounding.

Variance components of school performance		Raw estimate	Proportion of variance (95% CIs)
Causal due to self-control (SC)	b_SP,SC_^2^*(σ^2^_Asc_ + σ^2^_Dsc_ + σ^2^_Esc_)	0.333	0.0284 (0.019–0.051)
Causal due to grit	b_SP,Grit_^2^*(σ^2^_AGrit_ + σ^2^_DGrit_ + σ^2^_EGrit_)	0.032	0.0027 (0.001–0.011)
Causal due to covariance SC-grit	2*b_SP,SC_*b_SP,Grit_*(σ_Asc,Grit_ + σ_Dsc,Grit_ + σ_Esc,Grit_)	0.148	0.0126 (0.007–0.208)
Confounding due to A	2*b_SP,SC_*σ_ASC,SP_ + 2*b_SP,Grit_* σ_AGrit,SP_	1.457	0.124 (0.101–0.134)
Residual (res) variance	σ^2^_Ares_ + σ^2^_Dres_ + σ^2^_Eres_	9.769	0.832 (0.816–0.868)
Total	σ^2^_SP_	11.739	1

The total explained variance of school performance is 16.8%, with by far the largest part (12.4%) due to genetic confounding. The causal effects account for 4.4% (i.e., see Table 4: 2.84% + 0.27% + 1.26%) of the school performance variance. The decomposition of the 4.4% reveals that self-control (2.84% of the 4.4%) is a stronger predictor than grit (0.27% of the 4.4%).

The results based on the causal regression model with A confounding differ appreciably from the phenotypic regression results both in terms of explained variance and in terms of the relative contributions of self-control and grit. In the phenotypic regression analyses, we found that self-control and grit accounted for 28.4% of the school performance variance, and we found that grit was the stronger predictor in terms of unique contributions (grit contributed 13%, self-control contributed 4.4% to the total of 28.4%). In the causal regression model with A confounding, we found that the total explained variance is lower at 16.8%: 4.4% due to the causal effects of self-control and grit, and 12.4% due to additive genetic confounding. In contrast to the phenotypic regression, the stronger predictor here is self-control. Due to the remarkably high genetic correlation between grit and school performance (0.996, as shown in Table 2), grit emerges as the more influential factor in the phenotypic regression model. This strong association primarily arises from the presence of A confounding, which is not accounted for in the phenotypic regression model. Consequently, grit emerges as the stronger predictor in this model. Once we account for A confounding, the predictive value of grit is greatly reduced, and self-control emerges as the stronger predictor. The difference in total explained variance (28.4% in Model 1 vs 16.8% in Model 4) is a consequence of the influence of A confounding on the regression coefficients. The regression coefficients in the phenotypic regression model (Model 1) are b_SP,SC_ = 0.191 and b_SP,Grit_ = 0.331, compared to b_SP,SC_ = 0.151 and b_SP,Grit_ = 0.047 in the causal regression model with A confounding (Model 4). The bias in the regression model is due to confounding, as explained in detail in the Supplementary Material.

## Discussion

We investigated the association between two non-cognitive skills (self-control and grit) and school performance. Demonstrating the direct regression relations, while taking into account genetic or environmental confounding, lends credence to the causal interpretation of the regression relations (for reviews relating specifically to the twin design, see Kohler et al.^18^; McAdams et al.^19^). The present results support the hypothesis that school performance is causally dependent on self-control and grit. However, we also found that additive genetic confounding made a relatively large contribution to the associations. This confounding is due to pleiotropy: genes that are common to all three variables, independent of the direct regression relationships. With respect to effect sizes, we found that in total 16.8% of the school performance variance was explained. Of this 16.8%, genetic pleiotropy accounted for 12.4% and the causal effects accounted for 4.4%.

An educational implication is that children who lack self-control and grit may face a double disadvantage. Firstly, their genetic predisposition for low self-control and grit coexists with a genetic predisposition for low school performance. The genetic pleiotropy that we showed suggests that certain genetic variants associated with lower self-control and grit also contribute to lower academic achievement, regardless of the direct relationship. Secondly, their low self-control and grit directly impact their school performance, as indicated by the direct regression relationship. Therefore, these children face challenges both due to the genetic factors influencing multiple traits and their own limited self-control and grit, which together contribute to their lower academic performance. However, bearing in mind that 83.2% (i.e., 100%‒16.8%) of the school performance variance was unexplained, we emphasize that there are many other factors that may offset both the direct relationship and the genetic confounding.

Interventions designed to enhance self-control and grit (with the objective of improving school performance) are based on the assumption that self-control and grit are causally related to school performance. The present results demonstrate the truism that correlation does not imply causation. Notably, the straightforward regression analysis (Model 1, above) showed that self-control and grit accounted for 28% of the school-performance variance, which corresponds to a multiple correlation of 0.53 (i.e., √.28). However, taking into account confounding, the explained variance due to the direct, putatively causal relations is 4.4%, that is, a multiple correlation of 0.21 (√.044). In addition, straightforward regression analysis (Model 1) may identify a predictor as important, but its importance may be due to confounding, rather than its causal influence. Specifically, in the straightforward regression analysis (Model 1), grit emerged as the stronger predictor. However, when correcting for genetic confounding (Model 4), we found that self-control was a stronger causal predictor. This difference is due to the high genetic correlation between grit and school performance (see Table 2).

Taken the present results, the causal effect size (4.4% of variance, a multiple correlation of 0.21) suggests that an intervention is likely to have a relatively small effect in the population studied here (i.e., 11.5–12.5-year-old children in the Netherlands). However, it is important to be aware that, on the one hand, intervention studies, and on the other hand causal observational studies (including the present study), answer distinctive questions. As a result, they may yield contrasting findings, despite both being concerned with causality. Observational studies that use causal-inference techniques provide insights into causal relations within natural settings, addressing the question of “what is”. Intervention studies essentially alter the natural setting to examine the effectiveness of specific interventions, addressing the question of “what could be” in the light of the interventions^29,35^. As these questions are distinct, so could be the answers. Viewed from the “what is” perspective of the present study, one would expect an increase in self-control of one standard deviation to result in an increase in school performance of about 0.21 standard deviation units (ignoring the role of grit, to ease presentation). The relative size of this effect is hard to judge in isolation, as it should be evaluated relative to the effect sizes of other interventions targeting non-cognitive skills. More importantly, the effect of an intervention, designed ultimately to improve school performance, is unlikely to focus exclusively on self-control and grit, and unlikely to result in an intervention effect on school performance that is only attributable to self-control and grit. A comprehensive understanding of what works in educational programmes requires well-designed intervention research, including, where possible, randomized controlled trials. Note that randomized controlled trials, theoretically, do not suffer from genetic or environmental confounding, as the random assignment to intervention and control groups controls for all forms of confounding. In practice, however, controlling for confounding in randomized controlled trials is partially mitigated through volunteer bias and selective dropout.

The present study has several limitations. It is based on the classical twin design, which is a design that relies on underlying assumptions. Testing these assumptions is beyond the scope of this paper. We therefore emphasize that the reliable inference of causal relations and the associated effect sizes requires the triangulation of results from different designs and datasets. Other causal-inference methods, which may be used, include instrumental variable regression^20,36^ and random-intercept cross-lagged panel modelling^37^ (Mulder & Hamaker, 2021), as well as (quasi-)experimental designs (for application to non-cognitive abilities and school performance, see Yeager et al.^38^; Diamond et al.^39^).

The present results are based on the hypothesis that self-control and grit influence school performance. We have not ruled out the possibility of reverse causation or bidirectional causation. The decision to adopt the present unidirectional causal model is based on the prevailing literature (as discussed in the introduction), which predominantly suggests causal effect of self-control and grit on school performance.

We acknowledge that our phenotypic measures are not optimal. The measures of self-control, grit, and school performance were based on teacher ratings. With respect to school performance, we note that, in a subsample of the present sample, the teacher ratings correlate 0.70 with scores on the nationally standardized test of educational achievement (i.e., the CITO test)^10^. Nonetheless, ideally one would include teacher ratings as well as objective measures^40^. With respect to the non-cognitive skills, the teacher ratings may be biased by the teacher’s awareness of students’ school performance. We recognize that such awareness may introduce a bias, and result in an overestimation of the correlation between non-cognitive skills and academic achievement. However, the inclusion of the random rater effect accounts for any source of bias originating in the teachers as raters (including halo effects).

The grit measure used here mainly captures the perseverance of effort aspect of grit, not the consistency of interest. However, the literature consistently demonstrates that the perseverance of effort aspect holds greater importance for academic outcomes^2,15,41,42^.

In summary, our study sheds light on the relationship between non-cognitive traits and school performance. We found that self-control and grit are causally related to school performance (accounting for 4.4% of the school performance variance), with self-control emerging as the stronger causal predictor. Equally importantly, we found that additive genetic confounding contributed greatly to the associations between self-control and grit and school performance. This finding demonstrates the importance of taking into account confounding (regardless of its source) in interpreting regression relationships as causal.

## Methods

### Participants

The sample consisted of children registered in the Netherlands Twin Register (NTR). The NTR collects data from twins, their parents, and their siblings. The data of the children include self-ratings and parental and teacher ratings^43–45^. The data for this study are teacher ratings of the grit, self-control and school performance in 11.5–12.5-year-old twins. First, the parents of these twins were asked for permission to contact the teachers. Twins could be either in the same class and share a teacher or be different classes and be rated by different teacher. The sample included 3837 complete pairs and 1054 incomplete pairs (i.e., data missing on one member). Data was available on 8728 individuals. The sample consisted of 1957 monozygotic and 2934 dizygotic twin pairs. To ascertain the zygosity of the same-sex twin pairs, a DNA or blood test was conducted for 32.2% of the pairs, while for the remainder, parents completed a questionnaire that contained items related to the twins’ resemblance. Based on this questionnaire, zygosity is correctly determined in more than 96% of cases^45^.

The data collection procedure was approved by the ethical committee (called ‘Vaste Commissie Wetenschap en Ethiek’) at the Vrije Universiteit Amsterdam (VCWE-2021-111). Parents provided written informed consent.

### Materials

#### Self-control

The measure of self-control was based on the teacher ratings. The teachers completed the 8 items of the Achenbach Self-Control Scale (ASCS^46^ in the ASEBA-TRF^47^). The response options of each item are 0 (*not true*), 1 (*somewhat or sometimes tr*ue), and 2 (*very true or often true*). If more than three items were missing the sum scores was coded as missing. If three or fewer items were missing, the missing items were imputed using the individual (twin) level mean of the available items^46^. Of the self-control sum scores, 34.5% were constructed with one to three mean imputed items, due to changes in the content of the ASEBA-TRF over the years. The scores were reverse-coded, so the total score ranged from 0-16, with higher scores indicating better self-control. The Cronbach’s alpha in this sample is 0.87.

#### Grit

The measure of grit was based on the following three items relating to the perseverance aspect of grit: *Compared to typical pupils of the same age,* (1) *how hard does he/she work*; (*2*) *how appropriately does he/she behave*, and (3) *how task-oriented is he/she*. The teachers rated the twins with respect to these items using on a 7-point Likert scale. The item scores were summed to create grit sum scores. If a single item was missing, the individual (twin) level mean of the two observed items scores was used in calculating the individual grit sum score. If more than one item was missing, the grit sum score was coded as missing. In our sample, 55.2% had a missing score on item 3, due to changes in the content of the surveys over the years. The grit sum scores ranged from 1–21, with higher scores indicating more grit. Cronbach’s alpha of the grit measure is 0.90.

From the point of view of face validity, the second grit item appears to be quite general. However, Kevenaar et al.^10^ demonstrated that this item highly correlates with the other two items (items 1 and 2 correlate 0.70, items 2 and 3 correlate 0.71, and items 1 and 3 correlate 0.83). Moreover, they showed that the predictive value for school performance is not driven by this one item.

#### School performance

The measure of school performance was the sum score of teacher ratings, on a five-point scale, of the twins’ math, reading, and literacy performance^24,48^. School performance scores ranged from 3 to 15, with higher scores indicating better school performance. If a single rating was missing the individual (twin) level mean of the other two ratings was used for the missing value. The school performance sum score was coded as missing if more than one rating was missing, which was the case for 22.9% in our sample. Regarding reliability, the items correlations are 0.73 for reading-literacy, 0.51 for reading-math, and 0.67 for literacy-math. The Cronbach’s alpha of the school-performance measure was 0.84. Regarding validity, the school-performance measure correlated 0.70 with the nationally standardised test of academic achievement (i.e., the CITO test, see Kevenaar et al.^10^).

#### Sex and socioeconomic status (SES)

The covariate sex was coded 0 for boys and 1 for girls. The covariate parental socio-economic status (SES) was determined by a combination of their parents’ occupation and education, as described in de Zeeuw et al.^28^). The SES variable was coded on a scale of 1 to 4, with 1 indicating low SES and 4 indicating high SES. Sex and SES were included in the twin model as fixed covariates. That is, we fitted the twin model, while simultaneously regressing self-control, grit, and school performance on these covariates.

#### Same/different teacher

Twins in the same class were rated by the same teacher, while twins in different classes were rated by different teachers. To account for systematic rater bias, we coded rater sharing as 1 (twins in the same class, and rated by the same teacher), or 0 (twin members in different classes, and so rated by different teachers). We included rater as a random effect, i.e., as a source of variance common to the variables, due to systematic rater bias.

### Statistical modelling

We modelled the data in R using the OpenMx library^49^. As shown below, the distributions of all three phenotypes display negative skewness, as a consequence of ceiling effects. This is most notable in the distributions of self-control and grit. We fitted the models using full information maximum likelihood estimation, assuming that the data follow a right-censored multivariate normal distribution (as in de Zeeuw et al.^28^). We took the censoring into account explicitly to avoid bias stemming from the apparent ceiling effects.

We first fitted the saturated model, which serves to obtain estimates of the 6 × 6 MZ and DZ covariance (and correlation) matrices, corrected for censoring, and corrected for the covariates. We subsequently fitted the following six models: (1) the standard phenotypic regression model (taking into account the clustering of twins in families); (2) the trivariate ADE model to estimate the 3;× 3 covariance matrices Σ_A_, Σ_D_, and Σ_E_; (3) the causal regression model as depicted in Fig. 2, without A, D, or E confounding (i.e., the model with the correlations associated with the dashed double-headed arrows fixed to zero); and models 4, 5, and 6, i.e., the causal regression model with A confounding (Model 4), D confounding (Model 5), or E confounding (Model 6). Model 1 produces results based on the regression of school performance of self-control and grit, as one would obtain them in a sample of unrelated children. Model 2 is a standard trivariate ADE twin model. This model does not include any regression analyses; it provides estimates of the 3 × 3 covariance matrices Σ_A_, Σ_D_, and Σ_E_, and serves as a baseline model to evaluate the fit of Model 3, and Models 4, 5, and 6, as these models are nested under Model 2. If there is no confounding and if the regression relations are causal, we expect Model 2 to produce regression results comparable to those of Model 3, and we expect Model 3 to fit well (compare to Model 2). In case of A confounding, for instance, we expect Model 3 to fit poorly (compared to Model 2), and we expect Model 4 (causal regression with A confounding to fit well compared to Model 2).

We conducted a total of six likelihood-ratio tests: the comparison of the causal regression model without confounding with the ADE model (one test with 4 degrees of freedom [*df*]); the comparison of the causal regression model with A, D, or E confounding with the ADE model (three tests, each with 2 *df*); and the test of the causal regression coefficients (two tests, each 1 *df*) in the ultimate model of choice. As we conducted 6 likelihood ratio tests (LRTs), we corrected our family-wise alpha level of 0.05 using the Bonferroni correction^50^. resulting in an alpha of 0.05/6 = ~0.008 for each LRT. As mentioned, in all models, we included sex and SES as fixed covariates. We included rater (teacher) as a random covariate, to accommodate covariance among the measures rising from rater bias^51^.

### Reporting summary

Further information on research design is available in the Nature Research Reporting Summary linked to this article.

### Supplementary information

Supplementary

Reporting Summary

## Data Availability

Requests for access to NTR data: please see the procedures outlined on the following website: https://ntr-data-request.psy.vu.nl/.

## Code availability

Part of the code is available in the Supplementary Materials. Additional code is available upon request.
