# Supplementary material for: The relationship of school performance with self-control and grit is strongly genetic and weakly causal
Source: NPJ Sci Learn. 2023 Dec 4;8:53. doi: 10.1038/s41539-023-00198-3 (PMC10696063; doi:10.1038/s41539-023-00198-3)
Supplement: Supplementary file 1 — Supplementary [file 41539_2023_198_MOESM1_ESM.docx]

# Supplementary Material

For the following paper:

Kevenaar*, van Bergen*, Oldehinkel, Boomsma & Dolan (2024). The relationship of school performance with self-control and grit is strongly genetic and weakly causal. *npj Science of Learning*

## Supplementary Methods

Abbreviations

SP School performance, a phenotypic observed variable, conveyed in a square (Fig 1)

SC Self-control, a phenotypic observed variable, conveyed in a square (Fig 1)

Grit Grit, a phenotypic observed variable, conveyed in a square (Fig 1)

A Additive genetic variable, a latent variable, conveyed in a circle (Fig 1)

D Dominance variable, a latent variable, conveyed in a circle in the path diagram (Fig 1)

E Unshared environmental variable, a latent variable, conveyed in a circle in the path diagram

(Fig 1)

This supplement provides an account of the bias in the regression model originating in additive genetic confounding. The model is depicted in Figure 1. This model includes the phenotypic regression of SP on SC and Grit. The additive genetic confounding is represented by the dashed double headed arrows. The confounding represents a source of association between SP and SC and between SP and Grit, which is independent of the direct, phenotypic regression relations (coefficients b_SP,SC_ and b_SP,Grit_).

**Supplementary Figure 1**


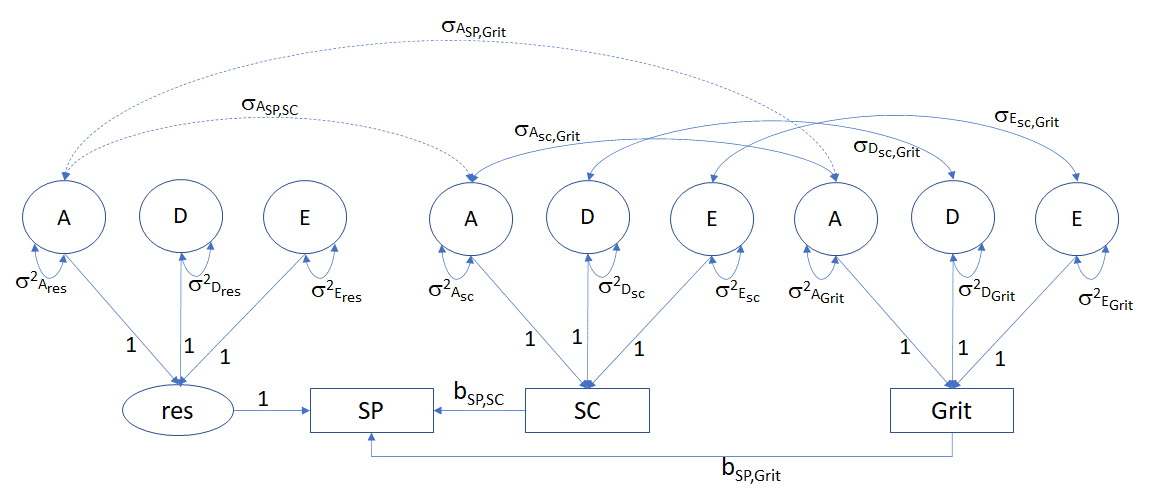


Given the model in Figure 1, we determine how the A confounding affects the phenotypic regression relationship given the following regression model (Figure 2):

**Supplementary Figure 2**


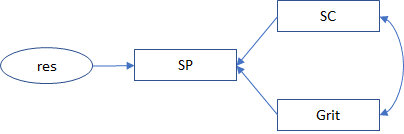


The model in Figure 1 can be fitted given twin data; the model in Figure 2 can be fitted given data obtained in unrelated individuals, as this is the straightforward regression of SP on SC and Grit. The results of fitting the modelling in Figure 2 are informative from a predictive point of view, but the relations cannot be interpreted causally. Any confounding will affect the explained variance of SP (R^2^) in the phenotypic regression model. This R^2^ could be completely due to confounding, in the most extreme case.

To determine how the A confounding (as shown in Fig 1) affects the results of the phenotypic regression analysis, we first determine the expected phenotypic covariance matrix associated with the “true” model (Figure 1),  This 3x3 covariance matrix  can be expressed as follows:

**** = (**I**-****)^-1^(**_A_** + **_D_** + **_E_**) (**I**-****)^-1t^, where

**_A_** = a11 **a21 a31** = σ^2^_Ares_ σ_ASP,SC_ σ_ASP,Grit_

**a21** a22 a32 σ_ASP,SC_ σ^2^_ASC_ σ_ASC,Grit_

**a31** a32 a33 σ_ASP,Grit_ σ_ASC,Grit_ σ^2^_AGrit_

where a21 and a31 are the source of confounding, i.e., a21 = σ_SP,SC_ and a31 = σ_SP,Grit_.

**_D_** = d11 0 0 = σ^2^_Dres_ 

0 d22 d32 ^2^_DSC_ σ_DSC,Grit_

0 d32 d33 _DSC,Grit_ σ^2^_DGrit_

**_E_**  = e11 0 0 = σ^2^_Eres_ 

0 a22 e32 ^2^_ESC_ σ_ESC,Grit_

0 e32 e33 _ESC,Grit_ σ^2^_EGrit_

(**I**-****) = 1 -bs -bg

0 1 0

0 0 1

(**I**-****)^-1^ = 1 bs bg

0 1 0

0 0 1,

where bs and bg are the regression coefficients (i.e., b_SP,SC_ and b_SP,Grit_, in Figure 2).

We represent the 3x3 covariance matrix  = **_A_**+**_D_**+**_E_** as follows:

*α β * = a11+d11+e11 a21 a31

*β  ε*  a21 a22+d22+e22 a32+d32+e32

* ε *  a31 a32+d32+e32 a33+d33+e33

We introduce the Greek notation merely to avoid clutter. The expected covariance matrix is  = (I-)^-1^ (I-)^-1t^ =

| *α* + bs**β* +bg** *+ bs*(*β *+ bs*** +bg** ε *) + bg*(* *+ bs**ε* +bg** *) | *β* + bs*** + bg**ε* | *χ* + bs**ε* + bg*** |
| --- | --- | --- |
| *β* + bs*** + bg**ε* | *δ* | *ε* |
| *χ* + bs**ε * + bg*** | *ε* | *φ* |

In fitting the linear regression model (Figure 2), we estimate the regression coefficients ***b***(2x1) as follows:

***b***= **_X_**^-1^**_XY_**, where 

**_X_=*** ε =* a22+d22+e22 a32+d32+e32

*ε * a32+d32+e32 a33+d33+e33

and

**_XY_** = *β *+ bs*** + bg**ε*

** + bs**E* + bg***

The inverse **_X_**^-1^ equals

**/(*δ* ** - ε ^2^*) *-ε*/(*δ* ** - ε ^2^*)

*-ε*/(*δ* ** - ε ^2^*) *φ*/(*δ* ** - ε ^2^*)*,*

where (* * – ε^2^*) is the determinant of **_X_**. Let **_X_**^-1^ equal

** /(*δ* **- ε ^2^*) *-ε*/(*δ* ** - ε ^2^*) *=* x11 x21

*- ε* /(*δ* ** - ε ^2^*) *φ* /(*δ* ** - ε ^2^*) x21 x22

So, we can express the regression coefficients in the phenotypic regression model (Fig 2) as follows:

***b*** =

x11*(*β *+ bs ** * +bg**ε *) + x21*(* *+ bs**ε* + bg***)

x21*(*β *+ bs ** * +bg**ε*) + x22*(* *+ bs**ε * + bg***)

***b*** =

** /(*δ* *** -* ε* ^2^)* (*β * + bs*** + bg**ε*) + (-*ε* /(*δ* ** * -* ε* ^2^))*( * * + bs**ε* + bg***)

(-*ε* /(*δ* *** -* ε* ^2^))*(*β* + bs*** + bg**ε*)+ * */(*δ* *** -* ε* ^2^)*(* * + bs**ε* + bg***)

We know that the A confounding is due to *β* =a21 and *χ* =a31. So, making this substitution, we have (conveying a21 and a31 in green to high these parameters):

***b*** =

x11*(a21+bs*** + bg**ε*) + x21*(a31+bs**ε* + bg***)

x21*(a21+bs*** + bg**ε*) + x11*(a31+bs**ε* + bg***)

The a21 and a31, being positive (see main article), result in an upwards bias in the regression estimates. Given a21=a31=0, we have

x11*(bs** * + bg**ε*) + x21*(bs**ε* + bg***)

x21*(bs** * + bg**ε*) + x11*(bs**ε* + bg***)

So, the regression coefficients in the phenotypic regression analysis (Figure 2) are overestimated by

x11*a21 + x21*a31 = *φ* /(*δ* *** -* ε* ^2^)*a21 + (-*ε* /(*δ* *** -* ε* ^2^))*a31

and

x21*a21 + x11*a31 = (-*ε* /(*δ* *** -* ε* ^2^))*a21 + *φ*/(*δ* *** -* ε* ^2^)*a31

The overestimation results in upward biased estimate of the proportion of explained variance in the phenotypic regression model.

**Numerical results**

We illustrate this numerically using the results as obtained. The values of bs and bg in the causal + A confounding model (Figure 1):

> print(c(bs, bg))

[1] 0.151 0.047

These values maximum likelihood estimates taken from the OpenMx output. We calculate these values based on the above, and obtain about the same results:

> print(c(f1_,f2_))

[1] 0.15100000 0.05110704

So ~.15 (self-control) and ~.05 (grit) are the values of the regression coefficients in the true model (Figure 1)

The values as calculated in the phenotypic regression model (Figure 2) are shown below (based on OpenMx output):

> print(c(f1,f2))

[1] 0.1906227 0.3307272

The values based on the above are about the same:

> print(c(g1,g2))

[1] 0.2067383 0.3123943

The bias in the regression coefficients due to confounding.

**> print(c(bias1, bias2))**

**[1] 0.05573831 0.26128727**

This corresponds to

x11*a21 + x21*a31 = *φ* /(*δ* *** -* ε* ^2^)*a21 + (-*ε* /(*δ* *** -* ε* ^2^))*a31

and

x21*a21 + x11*a31 = (-*ε* /(*δ* *** -* ε* ^2^))*a21 + *φ*/(*δ* *** -* ε* ^2^)*a31

The confounding is the source of the bias. In the true model (Figure 1), the regression coefficients are about .151 (SC) and .05 (Grit). In the regression model (Figure 2), we obtain about .20 (SC) and .32 (Grit). The differences about .151 vs .20 and .05 vs .32 is due to confounding (i.e., parameters a21 and a32, or, as conveyed in Figure 1, the covariances σ_ASP,SC_ andσ_ASP,Grit_).
